# Canopy Light Quality Modulates Stress Responses in Plants

**DOI:** 10.1016/j.isci.2019.11.035

**Published:** 2019-11-25

**Authors:** Sarah Courbier, Ronald Pierik

**Affiliations:** 1 Plant Ecophysiology, Department of Biology, Utrecht University, Padualaan 8, 3584 CH Utrecht, The Netherlands

**Keywords:** Biological Sciences, Plant Biology, Interaction of Plants with Organisms, Plant Physiology

## Abstract

Plants growing at high density are in constant competition for light with each other. The shade avoidance syndrome (SAS) is an effective way to escape neighboring vegetation. Even though the molecular mechanisms regulating SAS have been long studied, interactions between light and other environmental signaling pathways have only recently received attention. Under natural conditions, plants deal with multiple stresses simultaneously. It is, therefore, key to identify commonalities, distinctions, and interactions between plant responses to different environmental cues. This review outlines the current understanding of the interplay between canopy light signaling and other stresses, both biotic and abiotic. Understanding plant responses to multiple stimuli, factoring in the dominance of light for plant life, is essential to generate crops with increased resilience against climate change.

## Graphical Abstract

**Figure:**
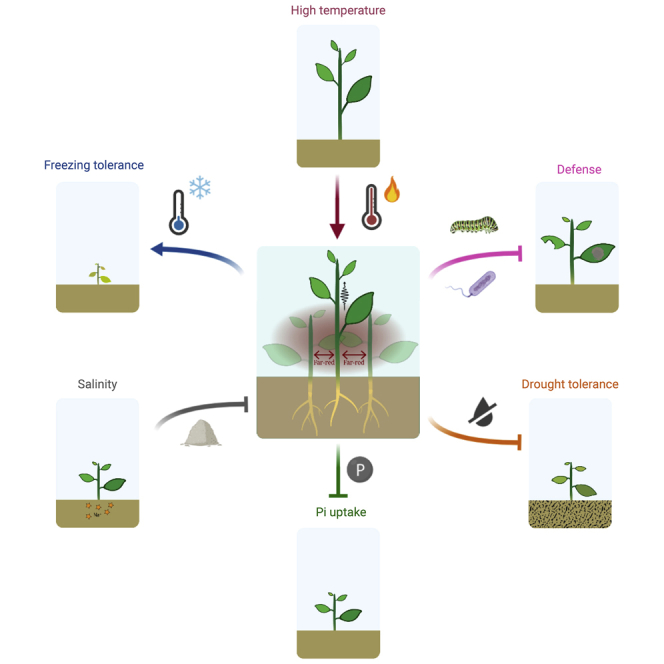

## Introduction

Global warming and overall climate change are major factors influencing plant life and fitness on the planet. Plants are increasingly challenged by their environment and they are forced to acclimate to sustain their survival. Also, as world population increases, food security is threatened by limited and continuously deteriorated arable land areas. The need for more resilient plants is, therefore, indispensable to feed the population in the future and one option is to grow plants at higher densities while maintaining individual plant productivity. The latter is not always straightforward because with increasing plant density, competition for light between individual plants increases as well, leading to growth reductions. Plants employ a suite of developmental adjustments to increase their competitive ability, known as the “shade avoidance syndrome (SAS)” (Ballaré and Pierik, 2017, Casal, 2013). SAS response is constituted of rapid hypocotyl, internode, and petiole elongation as well as upward movement of leaves (hyponasty), apical dominance and early flowering in Arabidopsis (Casal, 2012, Roig-Villanova and Martínez-García, 2016). Although immense progress has been made in unraveling the molecular mechanisms underpinning different SAS components, the impacts of density light cues and SAS responses on the ability of plants to deal with other challenging conditions such as abiotic and biotic stresses has not been very intensively studied. With the current knowledge on plant responses to single stresses, it becomes increasingly feasible to investigate if and how different stress responses are interconnected. This is of great importance since real-life conditions typically consist of multiple challenges occurring simultaneously. Because nearly all of these occur at high planting densities, especially in agricultural settings, we outline here the current knowledge on how light-mediated density responses interact with plant responses to (a)biotic stress. First, we briefly summarize the molecular and physiological mechanisms that plants deploy to control shade avoidance phenotypes. Then, we discuss the impact on responses to abiotic stresses such as salinity, drought, temperature, and submergence. We then discuss how responses to biotic stresses, covering both pathogenic and beneficial plant-microbe interactions, are modulated by density light signals. Lastly, we propose new research perspectives and directions toward a better understanding of plant responses to combined stresses in order to target breeding efforts and increase plant resilience to stresses at high planting density.

### Shade Avoidance Syndrome

**Figure 1:**
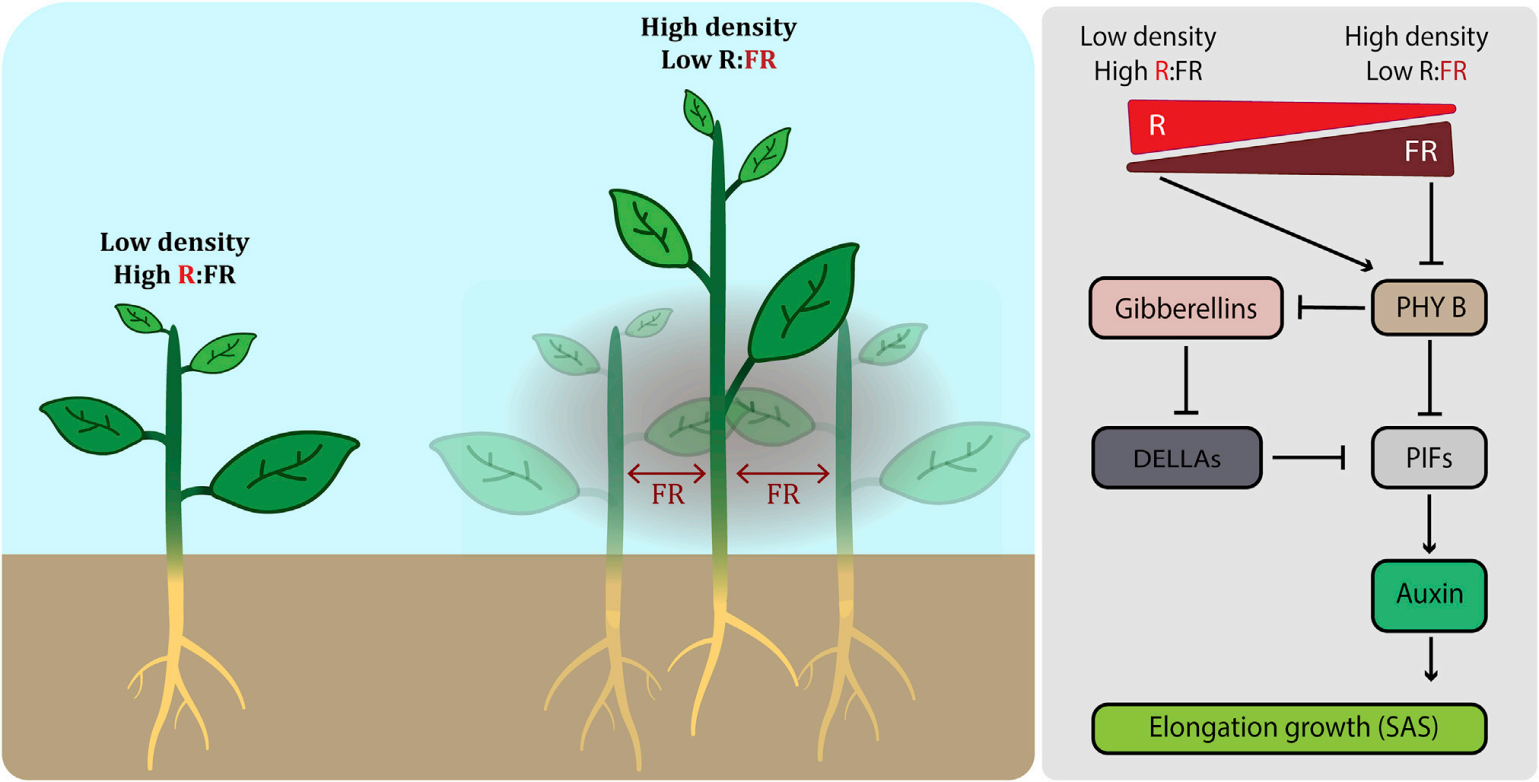
Simplified Overview of the Shade Avoidance Syndrome High planting densities lead to a reduction of the ratio between red and far-red light (R:FR), resulting in shoot elongation growth and slightly reduced root development. At the molecular level, low R:FR conditions result in reduced activity of phytochrome B (phyB). Inactive phyB allows phytochrome interacting factors (PIFs) to induce shoot elongation in an auxin-dependent manner. In parallel, gibberellin (GA) levels are increased, leading to the inhibition of the growth repressors, DELLA proteins, also leading to shoot elongation.

Plants need light to grow and have evolved elaborate light quality radars referred to as photoreceptors. Phytochromes (PHY) are sensitive to red (R) and far-red (FR) light; cryptochromes (CRY) and phototropins absorb ultraviolet (UV) A and blue (B) light, and these together are the long-established photoreceptors used by plants to sense their light environment (Quail, 1994). In addition, plants express the zeitlupe family of blue light receptors, ZTL, FKF1, and LKP2, (Demarsy and Fankhauser, 2009) and the UVR8 receptor for UV-B light detection (Rizzini et al., 2011). Plants absorb R and B light with chlorophyll in their chloroplasts for photosynthesis and reflect or transmit FR light. At high planting density, the absorption of R light and reflection of FR by plants lead to low Red: Far-red (R:FR) light conditions within the canopy (Figure 1). Changes in the R:FR ratio are sensed by phytochromes where phyB plays a major role in triggering shade avoidance in Arabidopsis (Franklin, 2008). Upon approximately equal fluence rates of R and FR (high R:FR), such as in direct sunlight, active phyB (Pfr) translocates to the nucleus, where it physically interacts with the bHLH transcription factors phytochrome interacting factors (PIFs) and promotes their inactivation and degradation by the 26S proteasome (Li et al., 2016). On the contrary, as planting density increases, R:FR decreases, and under such low R:FR, phytochrome is photoconverted into its inactive form (Pr). This inactivation disables PHY interaction with PIFs. Subsequently, PIFs can accumulate, bind G and E boxes in the promoters of their target genes to regulate their expression (Casal, 2013, Franklin, 2008, Franklin and Quail, 2010, Lorrain et al., 2008). Core targets for PIFs are genes associated with auxin homeostasis and cell wall remodeling to control growth (Figure 1) (Hornitschek et al., 2012, Li et al., 2012, Pedmale et al., 2016). Because auxin is mobile and its transport is highly coordinated in plants, this hormone also allows for coordination of growth responses across an organ or even organism in response to heterogeneous light conditions (Küpers et al., 2018, Pantazopoulou et al., 2017). Elongation growth depends on a key regulatory hub between brassinosteroids (BR), auxin, and gibberellin (GA) signaling pathways known as the BAP/D module that has PIFs at the core (Franciosini et al., 2017). This module is composed of three transcription factors BRASSINAZOLE-RESISTANT1 (BZR1), AUXIN RESPONSE FACTOR6 (ARF6), and PHYTOCHROME INTERACTING FACTOR4 (PIF4) that have been shown to physically bind each other to promote elongation growth and share half of their downstream targets (Oh et al., 2014). Growth promotion through the BAP/D module is inhibited by the negative growth regulators DELLAs. DELLAs have been shown to compete with the other transcription factors to bind ARF6 and to interact with PIFs and prevent their binding to target sequences on the DNA (Oh et al., 2014). However, upon low R:FR conditions, GA signaling is upregulated resulting in enhanced levels of bioactive GA, which subsequently leads to degradation of DELLAs, thus de-repressing PIFs (Li et al., 2016). Also, the inhibition of phyB by low R:FR contributes to release more PIFs to activate downstream growth-related gene expression (Figure 1).

Phytochrome signaling has also been shown to regulate the plant metabolic state associated with biomass production in Arabidopsis. Mutant plants lacking phyB showed a reduced chlorophyll content associated with lower CO_2_ uptake in leaves compared with the wild-type (WT) and pointing toward an impairment of photosynthesis (Yang et al., 2016). Even though photosynthesis might be impaired, *phyB* mutants accumulate more sucrose and starch in daytime but have reduced biomass compared with WT, indicating that phytochrome signaling plays a key role in biomass production and carbon resource allocation in Arabidopsis (De Wit et al., 2018, Yang et al., 2016). Interestingly, even though most plant species show a shade avoidance behavior (Roig-Villanova and Martínez-García, 2016), shoot elongation is not always an option. For example, plant species growing under tree canopies can obviously not outgrow the trees (Gommers et al., 2013). Recent studies in shade-tolerant and shade-avoiding *Geranium* species showed that shoot elongation upon low R:FR exposure only occurs in the grassland species *Geranium pyrenaicum* and not in *Geranium robertianum* mainly found in forest understories (Gommers et al., 2017). The mechanism behind shade tolerance is still poorly understood but could be of great importance in crop systems where an excessive elongation growth due to high plant density impairs yield and stem sturdiness in maize for example (Wang et al., 2016). Indeed, modification of the core, PIF-dependent SAS pathway seems to act in SAS suppression in a shade-tolerant plant (Gommers et al., 2018, Gommers et al., 2017). In addition to affecting shoot elongation, phyB signaling has also been reported to influence root development in an auxin-dependent manner (Figure 1) (Morelli and Ruberti, 2000). Low R:FR conditions sensed by phyB mainly in the shoot is sufficient to modulate auxin flow and gradients in the roots and control root growth in Arabidopsis (Salisbury et al., 2007). Upon additional FR radiation on Arabidopsis shoot, plants exhibit pronounced reductions in main root length and lateral root formation (van Gelderen et al., 2018). Low R:FR exposure in shoots stimulates the expression and stability of the transcription factor HY5, which can translocate from the shoot to the root system, and controls lateral root formation (van Gelderen et al., 2018). Even though shade avoidance is well understood in Arabidopsis, some aspects such as shade tolerance or shoot to root signaling have only recently been studied at mechanistic levels and are therefore still rather poorly understood.

## Shade Avoidance Interacts with Abiotic Stress Responses

Global food security is continuously threatened by weather fluctuations that can lead to drought or flooding events that severely affect crop yield. Climate change is associated with an increase in temperature and in the frequency of drought and flooding events over the world (Hirabayashi et al., 2013, Karl and Trenberth, 2003). Human-induced soil degradation is another factor influencing plant growth which is based on a proper uptake of nutrient and where imbalances can lead to stress responses. Although plant responses to abiotic stress are well studied, most studies are done on isolated plants, whereas crops typically grow at high density. Here, we will discuss the state of knowledge on how abiotic stress responses interact with R:FR-dependent shade avoidance and vice versa.

### Shade Avoidance and Soil-Borne Abiotic Stresses

**Figure 2:**
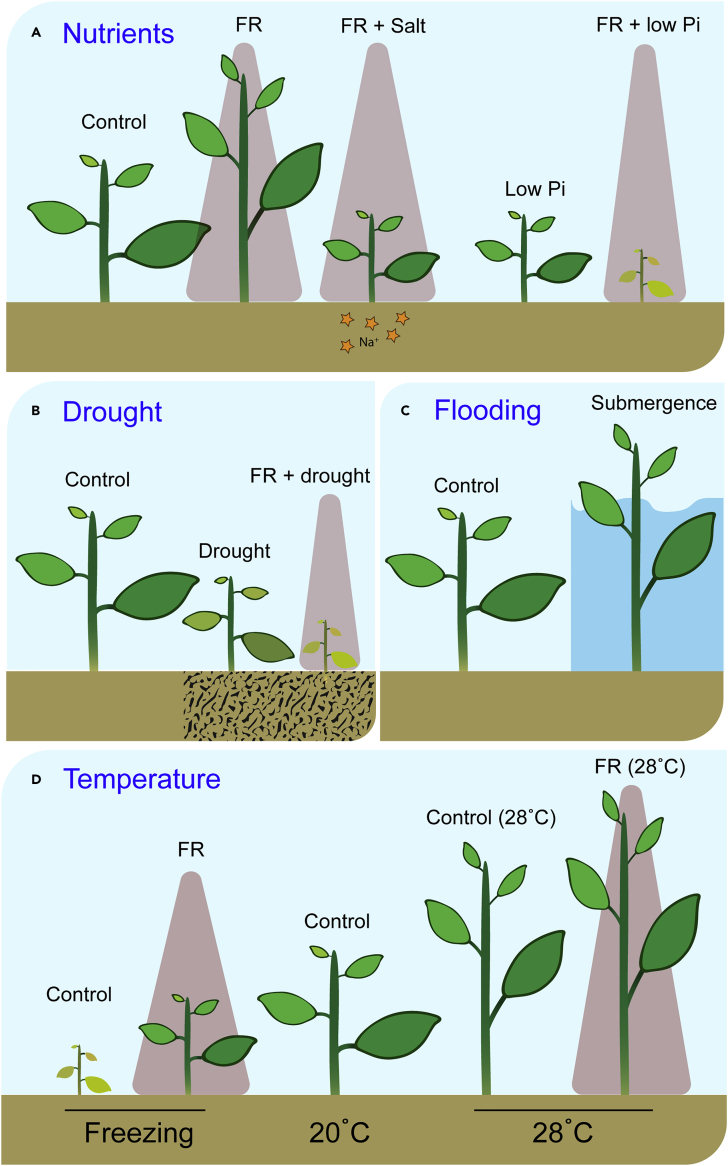
Shade Avoidance Affects Other Morphological Plant Responses Far-red (FR) modifies plant responses to abiotic stresses impacting their morphological responses to (A) nutrient availability, (B) drought, or (C) flooding stresses as well as (D) temperature changes.

Human agriculture has severely affected soil quality via irrigation, the use of fertilizers, and poor water drainage, which causes soil salinization as well as long-term nutrient deprivation, which is detrimental for plant development and yield. Interestingly, Arabidopsis roots are able to sense salinity and change their growth trajectory to grow away from high salt concentrations in the soil (Galvan-Ampudia et al., 2013). However, aspects of root architecture development, such as main root length and lateral root number and density are severely reduced by salinity (Dinneny et al., 2008, Julkowska et al., 2014, Pierik and Testerink, 2014). In addition to roots, also shoot growth and development are severely affected by soil salinity, for example, because of Na^+^ ion toxicity or osmotic stress resulting from saline conditions (Munns and Tester, 2008). Recently, Hayes et al. (2019) have shown that soil salinity inhibits SAS-associated hypocotyl and petiole elongation in Arabidopsis, as well as in several other species (Figure 2A). They found that ABA and BR signaling in Arabidopsis seedlings are responsive to salt and suppress shade avoidance. In low R:FR conditions, the inhibition of phyB allows PIF4 to induce the expression of *BR-Signaling Kinase 5 (BSK5)* having a key role in promoting the formation of a complex between PIF4 and the positive regulator of BR signaling BES1. In turn, the PIF4-BES1 complex is able to promote shoot elongation in an auxin-dependent manner (Hayes et al., 2019). Upon salt stress, ABA signaling leads to the inhibition of *BSK5* expression subsequently blocking the BES1-PIF4 complex and inhibiting the FR-induced shoot elongation in Arabidopsis (Hayes et al., 2019). Interestingly, Sakuraba et al. (2017) reported that PIF4 accumulation can induce genes involved in salt stress tolerance such as *SAG29* and *ORESARA1* (*ORE1/ANAC092*), showing that there may also be feedback from the shade avoidance pathway toward salinity tolerance. Nevertheless, it remains to be investigated how this would regulate salinity tolerance. In tomato, salinity stress leads to a severe growth reduction. Surprisingly, when salinity stress co-occurs with low R:FR conditions, plants show a higher growth rate compared with low R:FR conditions alone showing the synergy between salt responses and shade avoidance (Cao et al., 2018). The effect of salinity on tomato growth was gone in a *phyB1* mutant confirming the interaction between both responses. In addition, salinity-induced reactive oxygen species (ROS) production was decreased and ROS scavenging enzymes activity was increased when plants also experienced low R:FR conditions suggesting a protective role of low R:FR in salinity stress tolerance in tomato (Cao et al., 2018).

Soil degradation due to intensive agriculture is also associated with nutrient deficiency, which severely affects plant growth and development. Recently, phosphate (Pi) deficiency responses were shown to be regulated in a phyB-mediated manner. Sakuraba et al. (2018) demonstrated that PIF4 and PIF5 were able to directly downregulate Pi starvation-related gene expression such as *PHL1* and the Pi transporter *PHT1*;*1*. However, *pif4pif5* double mutants had increased Pi uptake in low Pi conditions compared with WT (Sakuraba et al., 2018), indicating that native PIFs suppress phosphorus uptake. Thus, plants experiencing growth at high density (where PIFs are highly abundant) and low Pi levels would likely perform even worse than on Pi-deficient soil without competition because of the inhibitory effect of PIFs on Pi acquisition (Figure 2A).

Plant response to water shortage, drought, has also been described to involve part of the shade avoidance signaling machinery. Arabidopsis plants lacking phyB show a lower stomatal conductance compared with WT in well-watered conditions (González et al., 2012). Upon water shortage, *phyB* mutants were slower in closing their stomata compared with WT, leading to increased water loss indicating the positive role of phyB in drought tolerance via the regulation of stomatal opening (González et al., 2012). Upon water deficit in tomato, ABA sensitivity in the guard cells of stomata is increased upon the presence of DELLA proteins via the inhibition of GA biosynthesis interfering with ABA receptors, causing an earlier stomata closure in tomato (Nir et al., 2017). The positive effect of phyB and DELLAs on drought tolerance in plants would probably be reversed at high density where the perception of low R:FR conditions promotes phyB inactivation and DELLA degradation (Djakovic-Petrovic et al., 2007). The earlier mentioned inhibition of root development under low R:FR light would in addition reduce the capacity of plants to absorb water from the soil and might thus further increase plant osmotic stress under water limitations. Indeed, it has been proposed that water limitation can attenuate the benefits of shade avoidance under natural conditions (Figure 2B) (Huber et al., 2004).

### Shade Avoidance and Aboveground Abiotic Stresses

Heavy rainfalls can cause soil waterlogging and subsequent whole-plant flooding events. Under submergence conditions, CO_2_ and O_2_ availability becomes limited, leading to an energy crisis (Sasidharan et al., 2018). Flood-tolerant plants such as *Rumex palustris* have evolved an escape strategy using light signaling components, leading to a rapid petiole elongation and leaf hyponasty to reach the water surface (Figure 2C). Upon submergence, the volatile plant hormone ethylene passively accumulates inside tissues, which leads to the downregulation of ABA signaling, in turn promoting elongation growth via transcriptional control of classic shade response components such as *COP1*, *PIF*, and *HY5* orthologs (van Veen et al., 2013). The clearly regulated light response pathway during submergence was found to be specifically regulated by the volatile plant hormone ethylene entrapped inside these tissues. Interestingly, a related species, *Rumex acetosa*, does not show submergence escape but can still show shade avoidance under water in response to light cues (Pierik et al., 2011), suggesting that light response pathway is conserved, but its activation by ethylene has only evolved in specific species. Indeed, a study on hypocotyl elongation in Arabidopsis seedlings exposed to either shade or elevated ethylene, confirmed two independent upstream pathways to converge on a shared downstream module to control elongation growth (Das et al., 2016).

In addition to drought and submergence, global climate change strongly increases average temperatures (Karl and Trenberth, 2003). Temperature is a very strong determinant of plant growth and architecture, and very high temperatures causing heat stress can even be lethal (Quint et al., 2016). Plants exposed to high temperature exhibit hyponasty and petiole elongation, very similar to the phenotypes elicited by low R:FR exposure (Figure 2D), but at elevated temperature their function is thought to promote leaf cooling (Bridge et al., 2013, Park et al., 2019). A few years ago, phyB conversion from Pfr to Pr was shown to be promoted by elevated temperature and phyB was shown to be a *bona fide* thermosensor in Arabidopsis (Jung et al., 2016, Legris et al., 2016). Furthermore, *phyb* mutants exhibit exaggerated elongation growth, whereas plants containing a constitutively active phyB showed very little thermomorphogenesis response at high temperatures (Jung et al., 2016). Also, the inactivation of phyB by elevated temperature facilitates the formation of the PIF4-BES1 complex mentioned earlier, which is able to trigger auxin signaling and lead to shoot elongation (Martínez et al., 2018). So, both FR enrichment and elevated temperature can inactivate phyB, thereby activating the core downstream pathways toward shoot elongation and hyponasty (Figure 2D). Importantly, increased temperature induced by global warming is also predicted to strengthen shade avoidance–mediated growth responses (Romero-Montepaone et al., 2019), which stresses the relevance of investigating the connections between different environmental cues to better improve crop quality. If higher temperature would indeed increase shade avoidance responses such as shoot elongation, this exaggerated elongation of non-harvestable organs would probably go at the expense of yield. Interestingly, when temperatures change in the opposite direction, low R:FR exposure triggers very little shade avoidance but strongly promotes leaf size and biomass accumulation at 16°C, which is not seen at 20°C (Patel et al., 2013). Lowering temperatures even further to freezing typically kills Arabidopsis unless they have been preconditioned to non-freezing temperatures such as 4°C to acquire freezing tolerance (Figure 2D). Interestingly, when plants are pre-exposed to low R:FR conditions, freezing tolerance is acquired even at 16°C (Franklin and Whitelam, 2007), further corroborating the intricate interactions between light quality and temperature responses (Figure 2D).

## Shade Avoidance Shapes Plant-Biotic Interactions

Low R:FR conditions have been shown to downregulate plant defense pathways against pathogens and herbivores (see Ballaré, 2014 for review). However, plants also interact with beneficial microorganisms, both to boost their defenses, but especially also to acquire nutrients under stressful, nutrient-limited conditions. Here, we outline the current understanding on the interplay between light signaling and defense signaling against pathogenic and beneficial microbes. Unraveling these connections in detail is crucial to improve plant fitness and resistance at high planting density.

### Low R:FR Light Inhibits Plant Immunity

**Figure 3:**
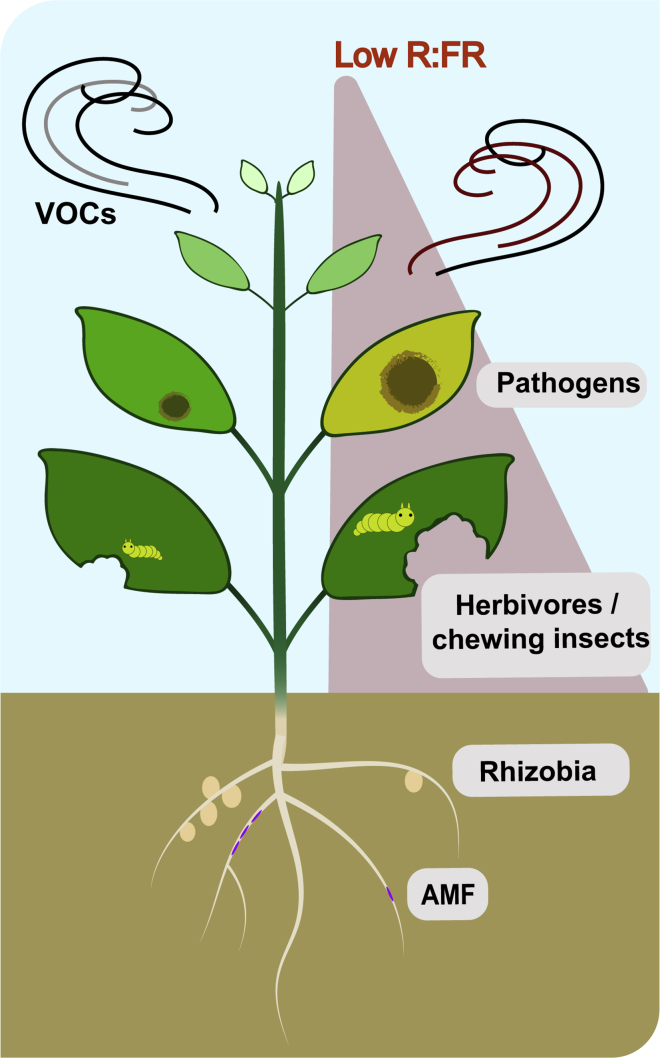
Low Red: Far-Red Modulates Plant Immunity Low R:FR increases susceptibility to pathogens and to chewing insects and shapes the plant volatiles organic compounds (VOCs) composition and become more attractive to herbivores. Upon perception of low R:FR conditions aboveground, plant root are less capable of forming nodules or arbuscules.

Plants carry an ever-evolving arsenal of defense mechanisms in order to restrict pathogens from colonizing (Jones and Dangl, 2006). Plants can recognize signatures from the attackers' surface such as pathogen-, microbe-, or damage-associated molecular patterns (PAMPs, MAMPs, and DAMPs), inducing rapid downstream defense signaling cascade referred as “PAMP-triggered immunity” (PTI) (Zipfel, 2014). The suppression of PTI by pathogen effectors or “effector triggered susceptibility” (ETS) is a sign of pathogen success that can be counteracted by plants recognizing those effectors and inducing the “effector triggered immunity” (ETI) (Cui et al., 2015, Peng et al., 2018) Plant defense responses against necrotrophic and biotrophic pathogens are mainly regulated by jasmonic acid (JA) and salicylic acid (SA), respectively (Glazebrook, 2005). The negative effect of low R:FR conditions on plant immunity has been in the research spotlight for the last decade and clear evidence of links between growth and defense mechanisms have been elucidated. JA is a core regulatory hormone orchestrating defense responses against insects and necrotrophic pathogens (Turner et al., 2013). Pioneering work demonstrated that plants lacking phyB or exposed to low R:FR conditions had decreased levels of resistance toward herbivores associated to reduced sensitivity to JAs (Figure 3) (Izaguirre et al., 2006, McGuire and Agrawal, 2005, Moreno et al., 2009). In Arabidopsis, low R:FR conditions reduce volatile organic compounds (VOCs) emissions as well as the induction of methyl-jasmonate (MeJA)-mediated gene expression upon herbivore attack (Kegge et al., 2013). Studies in crop systems such as tomato confirmed that low R:FR drastically modifies the MeJA-mediated VOCs composition and showed that this impacted indirect defenses by attracting predatory insects (Figure 3) (Cortés et al., 2016). In barley, constitutive VOCs emissions were altered in response to low R:FR and this was found to modulate plant-plant interaction responses (Kegge et al., 2015).

Several studies have addressed the mechanisms through which phytochromes would then modulate JA responses. Part of the mechanism appears to involve the interaction between the growth repressor DELLA proteins and the negative defense regulators JAZ proteins that are degraded upon JA signaling (Ballaré, 2014, Pieterse et al., 2014). During shade avoidance, DELLAs are degraded and this relieves their sequestration of JAZs. The release of JAZ proteins upon DELLA degradation leads to the inhibition of MYC2 transcription factor, which would otherwise activate downstream defense responses (Hou et al., 2010). Consistently, increased stability of JAZ10 was observed in the *phyB* mutant background in Arabidopsis, possibly following from DELLA degradation (Leone et al., 2014). Consistent with the importance of JAZ10, the hypersensitivity to *Botrytis cinerea* in the *phyB* mutant was partially overcome in the *jaz10 phyB* double mutant (Cerrudo et al., 2017).

Low R:FR conditions have been shown to downregulate JA-mediated, but also SA-mediated, defenses upon pathogen attack, coinciding with inhibition of NPR1 phosphorylation leading to poor downstream defense gene induction (De Wit et al., 2013). SA-deficient mutants were not able to fully exhibit petiole elongation response upon FR exposure, suggesting a role of SA in growth response in Arabidopsis (Nozue et al., 2018), that would need further studies to unravel the mechanisms. Interestingly, BR signaling that mostly regulates growth responses has been shown to be associated with flagellin (a well-known PAMP) recognition and signaling upon pathogen challenge via the interaction between the BR receptor kinase BRI1 and its coreceptor BAK1 (Chinchilla et al., 2007). BR is thought to inhibit defense responses through the induction of the positive growth regulator BRASSINAZOLE-RESISTANT 1 gene BZR1 (Lozano-Durán et al., 2013, Lozano-Durán and Zipfel, 2015). As mentioned earlier, BZR1 is a key component of the BAP/D module involved in growth regulation. Upon low R:FR conditions, plants might prioritize growth-mediated BR responses via the BAP/D module over flagellin-mediated defenses in case of a pathogen attack.

As previously mentioned, primary metabolism is strongly affected by low R:FR (De Wit et al., 2018, Yang et al., 2016). Carbohydrates are the main carbon source targeted by pathogens upon infection. The increased susceptibility observed in low R:FR or phytochrome mutant plants might also be associated with increased levels of accessible carbohydrates for the pathogen in plant tissue. High sugar levels in plants usually correlates with the production of secondary metabolites and/or defense gene induction such as MAPK, PR genes, or phenylpropanoid metabolic pathway (reviewed in Bolouri Moghaddam and Van den Ende, 2012). In low R:FR, reduced plant defense toward *B cinerea* was associated with reduced metabolite production and defense genes activation (Cargnel et al., 2014). Low R:FR thus seems to enhance soluble sugars and reduce defense-related processes that together would increase lesion development in pathogen-infected plant tissue. Although growth responses to shade cues seem to typically dominate investments into immune responses, the interactions are probably more complicated. It will be important to investigate if environmental conditions and developmental stage of the plant impact this crosstalk and perhaps even drive the direction of the interactions observed.

### Beneficial Interactions to Overcome Stressful Conditions

Plants do not only interact with pathogens but can also establish symbiotic interactions. One of the best studied is the interaction between legumes and the nitrogen-fixing Rhizobium-type bacteria that are able to colonize plant roots and form structures called “nodules” (Ferguson et al., 2010). Inside the nodules, N2 from the air can be fixed by Rhizobium into mineral nitrogen that can be used by the host plant. In return, carbohydrates generated by the plant are provided to the bacteria creating a situation where both host and symbiont benefit from the interaction (Ferguson et al., 2010). The *phyB* mutant of *Lotus japonicus* exhibits a constitutive shade avoidance phenotype just as in Arabidopsis but also has a reduced number of nodules compared with WT plants (Figure 3) (Suzuki et al., 2011). Grafting experiments showed that nodulation can be reduced in plants composed of *phyB* shoots and WT roots, indicating that the mutation in the shoot is sufficient to get the impoverished nodulation phenotype in the roots (Shigeyama et al., 2012, Suzuki et al., 2011). The nodulation deficiency in *phyB* mutants is associated with decreased root JA levels and a downregulation of JA-responsive gene expression compared with WT roots (Shigeyama et al., 2012, Suzuki et al., 2011). Interestingly, nodulation by *Bradyrhizobium japonicum* was promoted by end-of-day (EOD) low R:FR treatment in soybean (Balatti and Montaldi, 1986, Hunt et al., 1990), suggesting that there may be substantial species-specificity of the interplay between light and beneficial interactions.

Plants are also able to establish symbiotic interaction with phosphate-acquiring fungi known as “arbuscular mycorrhizal fungi” (AMF). These fungi are able to form highly branched structures within root cortical cells called “arbuscules” facilitating nitrogen and phosphate uptake from the soil. In return carbon-based supplies from the plant are directed to infected cortex cells and transformed into lipids by the plants before being transferred into the arbuscules (Keymer et al., 2017, Shachar-Hill et al., 1995). It was shown recently that low R:FR conditions reduce hyphal development of the AMF *Rhizophagus irregularis* in *Lotus japonicus* roots (Figure 3). This regulation is likely due to a downregulation of JA-responsive genes and reduced JA content in root exudates slowing down hyphal development of the fungus in the rhizosphere (Nagata et al., 2015, Nagata et al., 2016).

Symbiosis with both AMF and N2-fixing rhizobacteria may be under pressure during exposure to low R:FR light conditions that always occur at high plant density. Thus, when plants rely on these interactions because N or P availability in their soils is limited, the impact of this nutrient limitation may be increased by the presence of neighbor-derived FR light. The interplay between light and beneficial interactions is still unclear but further investigation into their mechanisms would help generate the knowledge needed to optimize crop plant growth, immunity, and yield at high planting densities in the future.

## Perspectives and Conclusion

Environmental abiotic and biotic stresses challenge plant growth, survival, and yield. Such stresses often occur simultaneously and it is key to understand the effect of sequential or combined stress responses in plants. Here, we have discussed how shade light signaling is mechanistically connected to other stress responses in Arabidopsis mainly. Overall, low R:FR conditions seem to mostly suppress adaptive responses to Pi deficiency, drought, pathogens as well as beneficial microbes in the soil (Figures 2 and 3). However, low R:FR enhances freezing tolerance, and key players of the shade avoidance pathway participate in plant elongation growth upon flooding stress. Other stresses such as salinity or elevated temperature are able to modulate low R:FR-mediated plant responses (Figure 2). In modern agriculture, plants are likely to be exposed at times to combinations of drought, nutrient deficiency, pathogenic, or beneficial microorganisms, and this might reduce growth capacity, defense, and yield if occurring at once. Also, as elevated temperatures strengthen plant growth responses to low R:FR, we expect that the negative impact of low R:FR on some environmental stresses may become worse as global temperatures increase. Unraveling the mechanisms of plant responses to simultaneous or sequential stresses in high density is therefore paramount to increase plant fitness and tolerance.

The well-established tradeoff between growth and defense is not so much an ON/OFF switch but appears to be a highly fine-tuned process (Ballaré and Austin, 2019). In crops, high-density-associated low R:FR conditions will suppress the JA pathways, which leads not only to a weakened defense pathway but also to a decrease in nodulation in legumes (Figure 3). It is paramount to understand how symbiotic and pathogenic interactions are affected by high density in order to create resilient plant systems in the future. Detailed studies on the molecular mechanisms of these interactions are required for this. There is indeed precedent that the pathways can be uncoupled, shown by specific mutants in Arabidopsis such as *sav3-2*, which lacks an elongation response, but still shows FR-mediated defense suppression (Cerrudo et al., 2012). Shade avoidance induction by blue light depletion rather than low R:FR on the other hand gives elongation without a measurable effect on resistance (Cerrudo et al., 2012). Forest understory plants are typically shade tolerant and show no shade avoidance responses to low R:FR. Interestingly, one such species, *G robertianum* does also not show downregulation by low R:FR of its JA-dependent defenses and even slightly increases its resistance against *B cinerea* (Gommers et al., 2017). Studies into the mechanisms have focused successfully on defense hormone signal transduction. An interesting novel avenue might be related to plant metabolism. Plants experiencing low R:FR or high-density growth conditions accumulate more soluble sugars during the day and synthesize fewer defense-associated secondary metabolites, possibly becoming better targets for pathogens. So far, it is unknown how changes in primary metabolism under low R:FR could affect plant disease resistance. Understanding how plant immune responses are modulated under shade conditions both in shade-tolerant and shade-intolerant plants might generate knowledge that can be translated toward crop improvement for growth at high planting density. Likewise, unraveling the molecular mechanisms underpinning interactions between photoreceptor signaling and abiotic stress pathways will provide the knowledge needed to optimize stress resilience in crops grown at the common-practice high planting densities.

**Figure 4:**
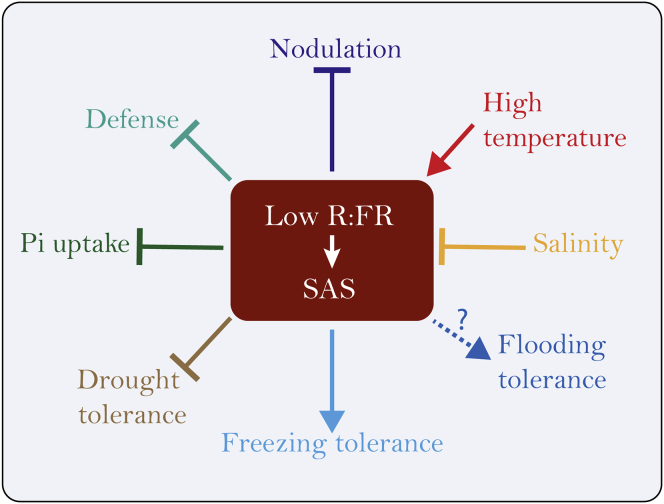
Summary of the Effect of Low R:FR Light Conditions on Other Stresses (Abiotic and Biotic)

In conclusion, we discussed here how low R:FR conditions or high planting densities impact stress responses in plants (Figure 4). We argue for more consideration of the planting density-associated light climate in studies on plant responses to (a)biotic stress. In addition, it turns out many response pathways are interconnected and their respective outputs at, for example, the phenotypic levels are likely co-regulated by these interconnections. We plea for integrative approaches to identify the core mechanistic nodes of these interactions that determine plant health and survival under naturally occurring (a)biotic stress dynamics, a topic of particular importance given the multitude of rapidly changing global climate conditions that threaten the global plant-based food production.

## References

[bib1] Balatti P.A., Montaldi E.R. (1986). Effects of red and far red lights on nodulation and nitrogen fixation in soybean (Glycine max L. Merr). Plant Soil.

[bib2] Ballaré C.L. (2014). Light regulation of plant defense. Annu. Rev. Plant Biol..

[bib3] Ballaré C.L., Austin A.T. (2019). Recalculating growth and defense strategies under competition: key roles of photoreceptors and jasmonates. J. Exp. Bot..

[bib4] Ballaré C.L., Pierik R. (2017). The shade-avoidance syndrome: multiple signals and ecological consequences. Plant Cell Environ..

[bib5] Bolouri Moghaddam M.R., Van den Ende W. (2012). Sugars and plant innate immunity. J. Exp. Bot..

[bib6] Bridge L.J., Franklin K.A., Homer M.E. (2013). Impact of plant shoot architecture on leaf cooling: a coupled heat and mass transfer model. J. R. Soc. Interface.

[bib7] Cao K., Yu J., Xu D., Ai K., Bao E., Zou Z. (2018). Exposure to lower red to far-red light ratios improve tomato tolerance to salt stress. BMC Plant Biol..

[bib8] Cargnel M.D., Demkura P.V., Ballaré C.L. (2014). Linking phytochrome to plant immunity: low red: far-red ratios increase Arabidopsis susceptibility to Botrytis cinerea by reducing the biosynthesis of indolic glucosinolates and camalexin. New Phytol..

[bib9] Casal J.J. (2013). Photoreceptor signaling networks in plant responses to shade. Annu. Rev. Plant Biol..

[bib10] Casal J.J. (2012). Shade avoidance. Arabidopsis Book.

[bib11] Cerrudo I., Caliri-Ortiz M.E., Keller M.M., Degano M.E., Demkura P.V., Ballaré C.L. (2017). Exploring growth-defence trade-offs in Arabidopsis: phytochrome B inactivation requires JAZ10 to suppress plant immunity but not to trigger shade-avoidance responses. Plant Cell Environ..

[bib12] Cerrudo I., Keller M.M., Cargnel M.D., Demkura P.V., de Wit M., Patitucci M.S., Pierik R., Pieterse C.M.J., Ballaré C.L. (2012). Low red/far-red ratios reduce Arabidopsis resistance to Botrytis cinerea and jasmonate responses via a COI1-JAZ10-dependent, salicylic acid-independent mechanism. Plant Physiol..

[bib13] Chinchilla D., Zipfel C., Robatzek S., Kemmerling B., Nürnberger T., Jones J.D.G., Felix G., Boller T. (2007). A flagellin-induced complex of the receptor FLS2 and BAK1 initiates plant defence. Nature.

[bib14] Cortés L.E., Weldegergis B.T., Boccalandro H.E., Dicke M., Ballaré C.L. (2016). Trading direct for indirect defense? Phytochrome B inactivation in tomato attenuates direct anti-herbivore defenses whilst enhancing volatile-mediated attraction of predators. New Phytol..

[bib15] Cui H., Tsuda K., Parker J.E. (2015). Effector-triggered immunity: from pathogen perception to robust defense. Annu. Rev. Plant Biol..

[bib16] Das D., St Onge K.R., Voesenek L.A.C.J., Pierik R., Sasidharan R. (2016). Ethylene- and shade-induced hypocotyl elongation share transcriptome patterns and functional regulators. Plant Physiol..

[bib17] De Wit M., George G.M., Ince Y.Ç., Dankwa-Egli B., Hersch M., Zeeman S.C., Fankhauser C. (2018). Changes in resource partitioning between and within organs support growth adjustment to neighbor proximity in Brassicaceae seedlings. Proc. Natl. Acad. Sci. U S A.

[bib18] De Wit M., Spoel S.H., Sanchez-Perez G.F., Gommers C.M.M., Pieterse C.M.J., Voesenek L.A.C.J., Pierik R. (2013). Perception of low red: far-red ratio compromises both salicylic acid- and jasmonic acid-dependent pathogen defences in Arabidopsis. Plant J..

[bib19] Demarsy E., Fankhauser C. (2009). Higher plants use LOV to perceive blue light. Curr. Opin. Plant Biol..

[bib20] Dinneny J.R., Long T.A., Wang J.Y., Jung J.W., Mace D., Pointer S., Barron C., Brady S.M., Schiefelbein J., Benfey P.N. (2008). Cell identity mediates the response of Arabidopsis roots to abiotic stress. Science.

[bib21] Djakovic-Petrovic T., de Wit M., Voesenek L.A.C.J., Pierik R. (2007). DELLA protein function in growth responses to canopy signals. Plant J..

[bib22] Ferguson B.J., Indrasumunar A., Hayashi S., Lin M.H., Lin Y.H., Reid D.E., Gresshoff P.M. (2010). Molecular analysis of legume nodule development and autoregulation. J. Integr. Plant Biol..

[bib23] Franciosini A., Rymen B., Shibata M., Favero D.S., Sugimoto K. (2017). Molecular networks orchestrating plant cell growth. Curr. Opin. Plant Biol..

[bib24] Franklin K.A. (2008). Shade avoidance. New Phytol..

[bib25] Franklin K.A., Quail P.H. (2010). Phytochrome functions in Arabidopsis development. J. Exp. Bot..

[bib26] Franklin K.A., Whitelam G.C. (2007). Light-quality regulation of freezing tolerance in Arabidopsis thaliana. Nat. Genet..

[bib27] Galvan-Ampudia C.S., Julkowska M.M., Darwish E., Gandullo J., Korver R.A., Brunoud G., Haring M.A., Munnik T., Vernoux T., Testerink C. (2013). Halotropism is a response of plant roots to avoid a saline environment. Curr. Biol..

[bib28] Glazebrook J. (2005). Contrasting mechanisms of defense against biotrophic and necrotrophic pathogens. Annu. Rev. Phytopathol..

[bib29] Gommers C.M.M., Buti S., Tarkowská D., Pěnčík A., Banda J.P., Arricastres V., Pierik R. (2018). Organ-specific phytohormone synthesis in two Geranium species with antithetical responses to far-red light enrichment. Plant Direct.

[bib30] Gommers C.M.M., Keuskamp D.H., Buti S., van Veen H., Koevoets I.T., Reinen E., Voesenek L.A.C.J., Pierik R. (2017). Molecular profiles of contrasting shade response strategies in wild plants: differential control of immunity and shoot elongation. Plant Cell.

[bib31] Gommers C.M.M., Visser E.J.W., Onge K.R.S., Voesenek L.A.C.J., Pierik R. (2013). Shade tolerance: when growing tall is not an option. Trends Plant Sci..

[bib32] González C.V., Ibarra S.E., Piccoli P.N., Botto J.F., Boccalandro H.E. (2012). Phytochrome B increases drought tolerance by enhancing ABA sensitivity in Arabidopsis thaliana. Plant Cell Environ..

[bib33] Hayes S., Pantazopoulou C.K., van Gelderen K., Reinen E., Tween A.L., Sharma A., de Vries M., Prat S., Schuurink R.C., Testerink C., Pierik R. (2019). Soil salinity limits plant shade avoidance. Curr. Biol..

[bib34] Hirabayashi Y., Mahendran R., Koirala S., Konoshima L., Yamazaki D., Watanabe S., Kim H., Kanae S. (2013). Global flood risk under climate change. Nat. Clim. Chang..

[bib35] Hornitschek P., Kohnen M.V., Lorrain S., Rougemont J., Ljung K., López-Vidriero I., Franco-Zorrilla J.M., Solano R., Trevisan M., Pradervand S. (2012). Phytochrome interacting factors 4 and 5 control seedling growth in changing light conditions by directly controlling auxin signaling. Plant J..

[bib36] Hou X., Lee L.Y.C., Xia K., Yan Y., Yu H. (2010). DELLAs modulate jasmonate signaling via competitive binding to JAZs. Dev. Cell.

[bib37] Huber H., Kane N.C., Heschel M.S., Von Wettberg E.J., Banta J., Leuck A.M., Schmitt J. (2004). Frequency and microenvironmental pattern of selection on plastic shade-avoidance traits in a natural population of impatiens capensis. Am. Nat..

[bib38] Hunt P.G., Kasperbauer M.J., Matheny T.A. (1990). Influence of Bradyrhizobium japonicum strain and far-red/red canopy light ratios on nodulation of soybean. Crop Sci..

[bib39] Izaguirre M.M., Mazza C.A., Biondini M., Baldwin I.T., Ballaré C.L. (2006). Remote sensing of future competitors: impacts on plants defenses. Proc. Natl. Acad. Sci. U S A.

[bib40] Jones J.D.G., Dangl J.L. (2006). The plant immune system. Nature.

[bib41] Julkowska M.M., Hoefsloot H.C.J., Mol S., Feron R., De Boer G.J., Haring M.A., Testerink C. (2014). Capturing arabidopsis root architecture dynamics with root-fit reveals diversity in responses to salinity. Plant Physiol..

[bib42] Jung J.H., Domijan M., Klose C., Biswas S., Ezer D., Gao M., Khattak A.K., Box M.S., Charoensawan V., Cortijo S. (2016). Phytochromes function as thermosensors in Arabidopsis. Science.

[bib43] Karl T.R., Trenberth K.E. (2003). Modern global climate change. Science.

[bib44] Kegge W., Ninkovic V., Glinwood R., Welschen R.A.M., Voesenek L.A.C.J., Pierik R. (2015). Red:far-red light conditions affect the emission of volatile organic compounds from barley (Hordeum vulgare), leading to altered biomass allocation in neighbouring plants. Ann. Bot..

[bib45] Kegge W., Weldegergis B.T., Soler R., Eijk M.V., Van Dicke M., Voesenek L.A.C.J., Pierik R. (2013). Canopy light cues affect emission of constitutive and methyl jasmonate-induced volatile organic compounds in Arabidopsis thaliana. New Phytol..

[bib46] Keymer A., Pimprikar P., Wewer V., Huber C., Brands M., Bucerius S.L., Delaux P.M., Klingl V., von Röpenack-Lahaye E., Wang T.L. (2017). Lipid transfer from plants to arbuscular mycorrhiza fungi. Elife.

[bib47] Küpers J.J., van Gelderen K., Pierik R. (2018). Location Matters: Canopy Light Responses over spatial scales. Trends Plant Sci..

[bib48] Legris M., Klose C., Burgie E.S., Rojas C.C.R., Neme M., Hiltbrunner A., Wigge P.A., Schäfer E., Vierstra R.D., Casal J.J. (2016). Phytochrome B integrates light and temperature signals in *Arabidopsis*. Science.

[bib49] Leone M., Keller M.M., Cerrudo I., Ballaré C.L. (2014). To grow or defend? Low red: far-red ratios reduce jasmonate sensitivity in Arabidopsis seedlings by promoting DELLA degradation and increasing JAZ10 stability. New Phytol..

[bib50] Li K., Yu R., Fan L.M., Wei N., Chen H., Deng X.W. (2016). DELLA-mediated PIF degradation contributes to coordination of light and gibberellin signalling in Arabidopsis. Nat. Commun..

[bib51] Li L., Ljung K., Breton G., Schmitz R.J., Pruneda-Paz J., Cowing-Zitron C., Cole B.J., Ivans L.J., Pedmale U.V., Jung H.S. (2012). Linking photoreceptor excitation to changes in plant architecture. Genes Dev..

[bib52] Lorrain S., Allen T., Duek P.D., Whitelam G.C., Fankhauser C. (2008). Phytochrome-mediated inhibition of shade avoidance involves degradation of growth-promoting bHLH transcription factors. Plant J..

[bib53] Lozano-Durán R., Macho A.P., Boutrot F., Segonzac C., Somssich I.E., Zipfel C. (2013). The transcriptional regulator BZR1 mediates trade-off between plant innate immunity and growth. Elife.

[bib54] Lozano-Durán R., Zipfel C. (2015). Trade-off between growth and immunity: role of brassinosteroids. Trends Plant Sci..

[bib55] Martínez C., Espinosa-Ruíz A., de Lucas M., Bernardo-García S., Franco-Zorrilla J.M., Prat S. (2018). PIF4-induced BR synthesis is critical to diurnal and thermomorphogenic growth. EMBO J..

[bib56] McGuire R., Agrawal A.A. (2005). Trade-offs between the shade-avoidance response and plant resistance to herbivores? Tests with mutant Cucumis sativus. Funct. Ecol..

[bib57] Morelli G., Ruberti I. (2000). Update on light signaling shade avoidance responses. Driving auxin along lateral routes. Plant Physiol..

[bib58] Moreno J.E., Tao Y., Chory J., Ballare C.L. (2009). Ecological modulation of plant defense via phytochrome control of jasmonate sensitivity. Proc. Natl. Acad. Sci. U S A.

[bib59] Munns R., Tester M. (2008). Mechanisms of Salinity Tolerance. Annu. Rev. Plant Biol..

[bib60] Nagata M., Yamamoto N., Shigeyama T., Terasawa Y., Anai T., Sakai T., Inada S., Arima S., Hashiguchi M., Akashi R. (2015). Red/far red light controls arbuscular mycorrhizal colonization via jasmonic acid and strigolactone signaling. Plant Cell Physiol..

[bib61] Nagata M., Yamamoto N., Miyamoto T., Shimomura A., Arima S., Hirsch A.M., Suzuki A. (2016). Enhanced hyphal growth of arbuscular mycorrhizae by root exudates derived from high R/FR treated Lotus japonicus. Plant Signal. Behav..

[bib62] Nir I., Shohat H., Panizel I., Olszewski N., Aharoni A., Weiss D. (2017). The tomato DELLA protein PROCERA acts in guard cells to promote stomatal closure. Plant Cell.

[bib63] Nozue K., Devisetty U.K., Lekkala S., Mueller-Moulé P., Bak A., Casteel C.L., Maloof J.N. (2018). Network analysis reveals a role for salicylic acid pathway components in shade avoidance. Plant Physiol..

[bib64] Oh E., Zhu J.Y., Bai M.Y., Arenhart R.A., Sun Y., Wang Z.Y. (2014). Cell elongation is regulated through a central circuit of interacting transcription factors in the Arabidopsis hypocotyl. Elife.

[bib65] Pantazopoulou C.K., Bongers F.J., Küpers J.J., Reinen E., Das D., Evers J.B., Anten N.P.R., Pierik R. (2017). Neighbor detection at the leaf tip adaptively regulates upward leaf movement through spatial auxin dynamics. Proc. Natl. Acad. Sci. U S A.

[bib66] Park Y.J., Lee H.J., Gil K.E., Kim J.Y., Lee J.H., Lee H., Cho H.T., Vu L.D., De Smet I., Park C.M. (2019). Developmental programming of thermonastic leaf movement. Plant Physiol..

[bib67] Patel D., Basu M., Hayes S., Majláth I., Hetherington F.M., Tschaplinski T.J., Franklin K.A. (2013). Temperature-dependent shade avoidance involves the receptor-like kinase ERECTA. Plant J..

[bib68] Pedmale U.V., Huang S.S.C., Zander M., Cole B.J., Hetzel J., Ljung K., Reis P.A.B., Sridevi P., Nito K., Nery J.R. (2016). Cryptochromes interact directly with PIFs to control plant growth in limiting blue light. Cell.

[bib69] Peng Y., Van Wersch R., Zhang Y. (2018). Convergent and divergent signaling in PAMP-triggered immunity and effector-triggered immunity. Mol. Plant Microbe Interact..

[bib70] Pierik R., De Wit M., Voesenek L.A.C.J. (2011). Growth-mediated stress escape: convergence of signal transduction pathways activated upon exposure to two different environmental stresses. New Phytol..

[bib71] Pierik R., Testerink C. (2014). The art of being flexible: how to escape from shade, Salt, and drought1. Plant Physiol..

[bib72] Pieterse C.M.J., Pierik R., Van Wees S.C.M. (2014). Different shades of JAZ during plant growth and defense. New Phytol..

[bib73] Quail P.H. (1994). Photosensory perception and signal transduction in plants. Curr. Opin. Genet. Dev..

[bib74] Quint M., Delker C., Franklin K.A., Wigge P.A., Halliday K.J., Van Zanten M. (2016). Molecular and genetic control of plant thermomorphogenesis. Nat. Plants.

[bib75] Rizzini L., Favory J.J., Cloix C., Faggionato D., O’Hara A., Kaiserli E., Baumeister R., Schäfer E., Nagy F., Jenkins G.I., Ulm R. (2011). Perception of UV-B by the arabidopsis UVR8 protein. Science.

[bib76] Roig-Villanova I., Martínez-García J.F. (2016). Plant responses to vegetation proximity: a whole life avoiding shade. Front. Plant Sci..

[bib77] Romero-Montepaone S., Poodts S., Fischbach P., Sellaro R., Zurbriggen M.D., Casal J.J. (2019). Shade-avoidance responses become more aggressive in warm environments. BiorXiv.

[bib78] Sakuraba Y., Bülbül S., Piao W., Choi G., Paek N.C. (2017). Arabidopsis EARLY FLOWERING3 increases salt tolerance by suppressing salt stress response pathways. Plant J..

[bib79] Sakuraba Y., Kanno S., Mabuchi A., Monda K., Iba K., Yanagisawa S. (2018). A phytochrome-B-mediated regulatory mechanism of phosphorus acquisition. Nat. Plants.

[bib80] Salisbury F.J., Hall A., Grierson C.S., Halliday K.J. (2007). Phytochrome coordinates Arabidopsis shoot and root development. Plant J..

[bib82] Sasidharan R., Hartman S., Liu Z., Martopawiro S., Sajeev N., Van Veen H., Yeung E., Voesenek L.A.C.J. (2018). Signal dynamics and interactions during flooding stress. Plant Physiol..

[bib83] Shachar-Hill Y., Pfeffer P.E., Douds D., Osman S.F., Doner L.W., Ratcliffe R.G. (1995). Partitioning of intermediary carbon metabolism in vesicular-arbuscular mycorrhizal leek. Plant Physiol..

[bib84] Shigeyama T., Tominaga A., Arima S., Sakai T., Inada S., Jikumaru Y., Kamiya Y., Uchiumi T., Abe M., Hashiguchi M. (2012). Additional cause for reduced JA-Ile in the root of a Lotus japonicus phyB mutant. Plant Signal. Behav..

[bib85] Suzuki A., Suriyagoda L., Shigeyama T., Tominaga A., Sasaki M., Hiratsuka Y., Yoshinaga A., Arima S., Agarie S., Sakai T. (2011). Lotus japonicus nodulation is photomorphogenetically controlled by sensing the red/far red (R/FR) ratio through jasmonic acid (JA) signaling. Proc. Natl. Acad. Sci. U S A.

[bib86] Turner J.G., Ellis C., Devoto A. (2013). The jasmonate signal pathway. Plant Cell.

[bib87] van Gelderen K., Kang C., Paalman R., Keuskamp D., Hayes S., Pierik R. (2018). Far-red light detection in the shoot regulates lateral root development through the HY5 transcription factor. Plant Cell.

[bib88] van Veen H., Mustroph A., Barding G.A., Vergeer-van Eijk M., Welschen-Evertman R.A.M., Pedersen O., Visser E.J.W., Larive C.K., Pierik R., Bailey-Serres J. (2013). Two Rumex species from contrasting hydrological niches regulate flooding tolerance through distinct mechanisms. Plant Cell.

[bib89] Wang H., Wu G., Zhao B., Wang B., Lang Z., Zhang C., Wang H. (2016). Regulatory modules controlling early shade avoidance response in maize seedlings. BMC Genomics.

[bib90] Yang D., Seaton D.D., Krahmer J., Halliday K.J. (2016). Photoreceptor effects on plant biomass, resource allocation, and metabolic state. Proc. Natl. Acad. Sci. U S A.

[bib91] Zipfel C. (2014). Plant pattern-recognition receptors. Trends Immunol..

